# High-performance Brillouin spectroscopy using VIPA-etalon cascades

**DOI:** 10.1117/1.JBO.31.2.026001

**Published:** 2026-02-03

**Authors:** Sophie Chagnon-Lessard, Julian Nicolai, Joshua Steller, Eng Kuan Moo, Hubert Jean-Ruel

**Affiliations:** a Carleton University, Department of Electronics, Faculty of Engineering and Design, Ottawa, Ontario, Canada; b Carleton University, Department of Mechanical and Aerospace Engineering, Faculty of Engineering and Design, Ottawa, Ontario, Canada

**Keywords:** Brillouin spectroscopy, contrast enhancement, biomedical applications, virtually imaged phased array, cascaded Fabry-Perot etalon, confocal collection, line-scanning

## Abstract

**Significance:**

Brillouin spectroscopy noninvasively probes mechanical properties of biological materials and has become a valuable tool in various areas of biomedical research. However, the limited contrast of conventional single stage virtually imaged phased array (VIPA) spectrometers restricts many applications. Current contrast enhancement methods have limitations, including incompatibility with line-scanning collection, which is highly advantageous for fast biomechanical mapping.

**Aim:**

The aim is to develop and characterize a high-throughput contrast enhancement technique using a VIPA-etalon cascade, designed for compatibility with both confocal and line-scanning geometries across a wide range of wavelengths.

**Approach:**

One or more etalons with approximately matched thicknesses are integrated downstream of the VIPA to suppress its Lorentzian tails. A theoretical model and ray-tracing simulation were developed, complemented by experimental validation in confocal and line-scanning setups at 785 and 532 nm, respectively. Brillouin spectra were measured for reference materials and biological samples, including a porcine crystalline lens.

**Results:**

Experimentally, the integration of a single high-finesse cascade yielded contrast enhancements of 27 and ∼19  dB for the confocal and line-scanning geometries, respectively, with less than 50% reduction in peak transmission in both cases. Simulations demonstrated that substantially higher contrast can be achieved in principle with negligible impact on throughput by serializing multiple low-finesse etalons. High-quality Brillouin spectra were obtained in both collection geometries, demonstrating robust performance across diverse samples.

**Conclusions:**

The VIPA-etalon cascade markedly improves the performance of Brillouin spectroscopy, offering a straightforward, cost-effective, and versatile solution for analyzing biological samples. It enables precise, high-resolution biomechanical investigations, advancing applications in biomedical research and clinical diagnostics.

## 1 Introduction

Brillouin spectroscopy has emerged as a powerful, noninvasive technique for probing the mechanical properties of biological materials.^1^^,^^2^ It offers critical insights into cellular processes and disease mechanisms for biomedical research, and its potential for use in diagnostics is currently an active area of research.^3^^–^^5^ For many applications—such as in the study of the viscoelastic properties of cells in neurodegenerative diseases,^6^ the elastic properties of cancerous tissue,^7^ or garnering insight into ocular health from biomechanical parameters^8^—the use of high-resolution 2D and 3D Brillouin imaging is invaluable. However, the slow acquisition speeds of traditional Brillouin systems often hinder practical implementation.

Virtually imaged phased array (VIPA) spectrometers were developed to address the limited acquisition speed of tandem Fabry–Perot (FP) interferometers, which paved the way for use in biological applications.^9^^–^^11^ Nevertheless, point-by-point mapping with the standard confocal geometry remains slow, and the spectral contrast of standard, single-stage VIPA spectrometers is insufficient for most samples. A number of contrast enhancement and notch filtering methods have been developed to improve spectral contrast, with each having different trade-offs.^9^^,^^12^^–^^19^ To address the speed limitation in mapping, parallel measurements via line-scanning acquisition were recently developed.^20^^–^^22^ However, this geometry is incompatible with most contrast enhancement methods, including the commonly employed multistage crossed VIPA configuration. Rubidium cell filtering has been the primary approach to circumvent the VIPA contrast limitation in line-scanning measurements, but it has certain limitations and constraints, including imposing the use of 780 nm excitation. Improved techniques for enhancing the contrast of VIPA spectrometers in confocal and line-scanning geometries are therefore needed.

To address this gap, we introduce and study a novel Brillouin spectrometer scheme utilizing a VIPA-etalon cascade configuration, which combines high contrast and throughput while maintaining compatibility across both confocal and line-scanning geometries at any wavelength. Computational simulations, experimental characterization, and reference Brillouin measurements are presented, demonstrating the approach’s potential for use in advanced biomedical applications.

## 2 Principle and Methods

### 2.1 Proposed Concept

When acquiring a Brillouin spectrum using a standard single-stage VIPA, the typically overwhelming elastic scattering often jeopardizes the extraction of the Stokes and anti-Stokes peaks in the following way. The VIPA’s Lorentzian tails produce lines of contaminating pump signal on the sensor between the elastic peaks (VIPA orders). Without a cross-disperser, the much weaker Brillouin peaks are buried below these lines, substantially reducing the signal-to-noise ratio or preventing detection altogether. In practice, this intrinsic limitation of VIPAs hinders measurements of nontransparent, highly scattering samples.

**Fig. 1:**
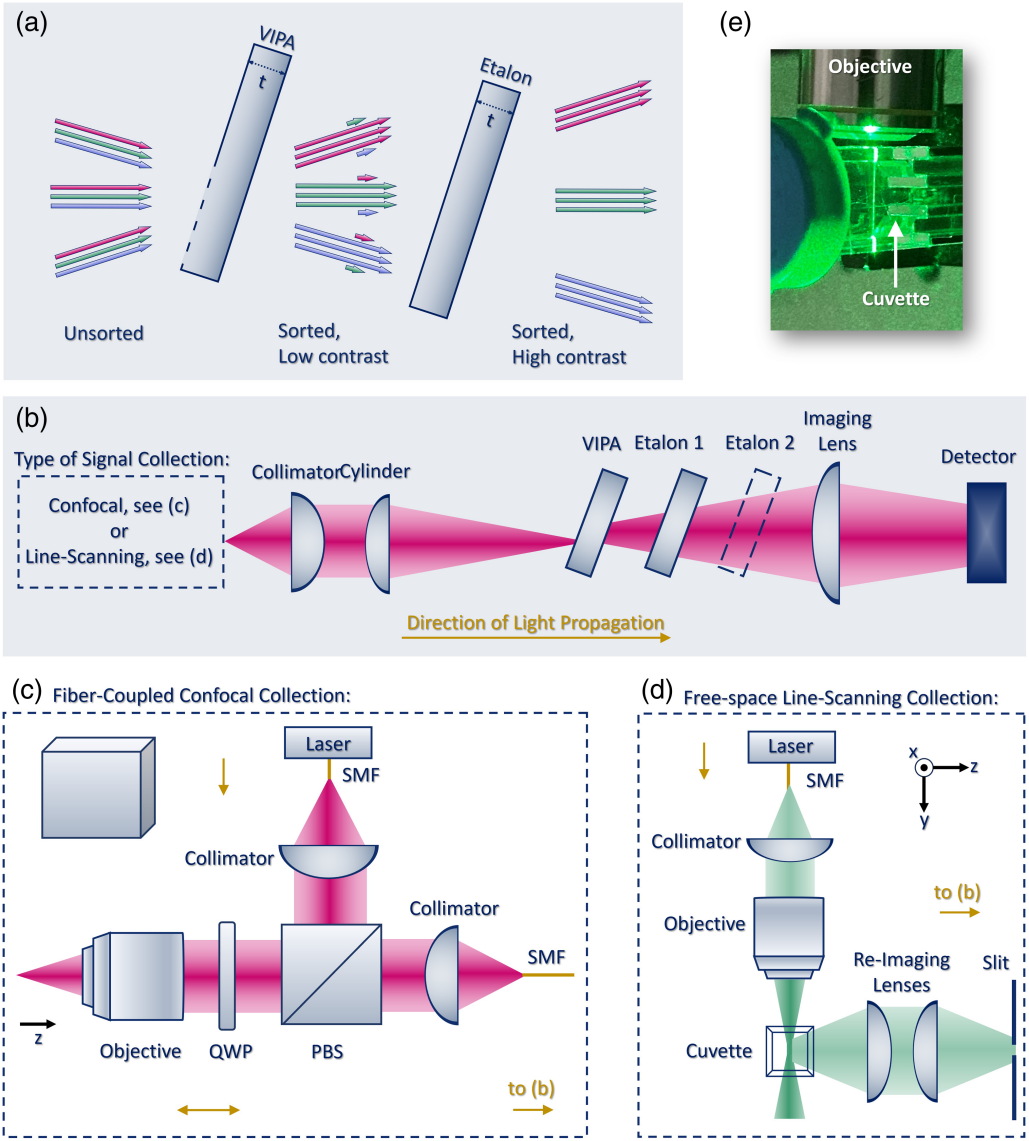
Proposed concept and configuration. (a) Depiction of the contrast enhancement with preserved throughput achieved by cascading one or multiple near thickness- and angle-matched etalons downstream of a VIPA. (b) Configuration of the Brillouin spectrometer. (c) Illustration of the confocal excitation and collection modality. (d) Illustration of the line-scanning excitation and collection modality. (e) Photograph of a cuvette filled with water installed in the line-scanning setup.

We propose a Brillouin spectrometer approach centered on a synergistic VIPA-etalon cascade that maintains high throughput while suppressing the unwanted Lorentzian tails. Figure 1(a) illustrates the strategy for achieving this high-performance configuration. The VIPA sorts the different wavelengths at different angles but is limited by an inherent contrast of 30 to 35 dB. To address this limitation, the VIPA is followed with one, two, or more approximately parallel-oriented standard Fabry–Perot etalons. For a given input angle, each etalon selectively transmits resonant wavelengths while filtering out nonresonant rays through back reflection. Unlike that of a VIPA—which typically achieves a throughput of ∼90%—the throughput of a single etalon is typically below 10% with cylindrically diverging light (∼1/finesse). Remarkably, the VIPA-etalon configuration transmits nearly all incident light. This high efficiency stems from precisely matching the VIPA and etalon dispersion, enabling their synergistic performance. By matching their thicknesses and tilt angles, the VIPA pre-sorts each wavelength at the specific angles of maximum etalon transmission.^23^ Simultaneously, the small but problematic fraction of unsorted wavelengths from the VIPA are filtered out by the etalon, greatly enhancing contrast.

### 2.2 Methods

#### 2.2.1 MATLAB model

A simple model based on standard FP theory^24^ was developed in MATLAB to simulate the response of the VIPA-etalon cascade. Analogous to Fig. 1, it consists of either a small-diameter Gaussian point source or a uniform line source for the confocal and line-scanning systems, respectively. The light source is first collimated (nominally) and then converged in the *y*-axis to uniformly illuminate an arbitrarily large range of angles. It is then made incident on a series of FP cavities before being focused on a sensor by an imaging lens.

To determine the final output image on the sensor, two independent maps must be calculated: the transfer function of the series of FPs and the normalized system response omitting the FP elements. First, the normalized transfer function of each FP is calculated (the VIPA, then each cascaded etalon) and multiplied together to form the total FP response. Each pixel on the sensor being associated with a specific angle of incidence through a given FP, the corresponding FP transmission (transfer function) can be calculated based on that incident angle θ, reflectance R, cavity length d, refractive index n, and source wavelength in vacuum λ:^24^

$$T_{\mathrm{FP}}=\frac{(1-R{)}^{2}}{1+R^{2}-2R\;\cos(4\pi nd\lambda^{-1}\cos\;\theta )}.$$ (1)

Equation (1) assumes there are no resonator losses. The incident angle depends on the imaging lens focal length and the FP tilt angle in both axes. To note, as the etalon cascade is relative to the VIPA output where only the normalized transfer function is desired, the VIPA may be treated as a standard etalon. Second, the normalized response of the system due to the source (sans FP) is determined. The source is effectively re-imaged along the *x*-axis (magnified by the focal length ratio of the imaging lens and collimator) while spread as a flat, uniform envelope along the *y*-axis. The Gaussian forms a uniform line in the *y*-axis, while the line source forms a uniform rectangle. The total FP transfer function is then applied to the system response (sans FP) to form the output image response.

The modeled contrast curves are wavelength-independent. In this example, a wavelength of 785 nm was used. The VIPA parameters were: 3.3700000 mm thickness, *y*-tilt of 2 deg, *x*-tilt of 0 deg, a refractive index of 1.45 (fused silica), and reflectances of 100% and 92% (mimicked with equal reflectances of $\sqrt{0.92}\times 100\%$ for convenience) for the front and back reflectors, respectively. The etalon parameters were similar except that the reflectance was 94% (confocal case and line-scanning case with single-stage cascade) or 75% (line-scanning case with dual-stage cascade) on both surfaces, the *x*-tilt was 0.1 deg, and the thickness was adjusted by a few nanometers (3.3700025 mm) to coincide with the VIPA and etalon orders.

#### 2.2.2 Ray tracing simulations

A ray tracing simulation was developed using Ansys OpticStudio (version 2024 R1.02) in non-sequential mode. A monochromatic 532 nm Gaussian point source with a numerical aperture (NA) of ∼0.1 is collimated using a 50 mm (confocal) or 75 mm (line-scanning) achromatic doublet. A 400 mm cylindrical singlet lens focuses the beam into a line along the x-axis, coupling into the entrance window of a fused silica VIPA tilted by 2 deg in the *y*-axis and 0° in the *x*-axis. The VIPA is constructed with two combined rectangular volumes: the lower entrance window and the upper interferometer, both with a thickness of 3.3700000 mm to produce a ∼30  GHz free spectral range (FSR). The reflectance of the back and front surfaces of the VIPA is 0% and 92%, respectively, for the lower section and 100% and 92% for the upper section. A fused silica etalon, tilted by 2 deg in the *y*-axis and 0.2 deg in the *x*-axis, is placed 5 mm downstream of the VIPA with a one-way absorbing plane in between to prevent unphysical artifacts. The etalon consists of a rectangular volume with surface reflectances of 94% and a thickness of 3.3700098 mm. The output of the cascade is focused using a 300 mm achromatic doublet lens onto a 1D detector.

#### 2.2.3 Confocal scheme and 785 nm spectrometer

The confocal scheme uses a 785 nm single longitudinal mode (SLM) laser diode (IPS, I0785SD0090BX-IS-HD), which is first coupled to a single-mode fiber (SMF) before being routed into the confocal microscope stage using a fiber collimator. The beam then passes through a polarizing beam splitter (PBS) and a quarter waveplate (QWP), which is focused inside the sample with an underfilled, 0.25 NA, 10× objective. The laser power was set to 30 mW at the sample plane. The back-reflected elastic and Brillouin signals pass back through the objective and QWP before being reflected by the PBS, which redirects the light to a collimator for fiber-coupling to the spectrometer. The spectrometer first collimates the light using a 50 mm achromatic doublet and then focuses it into the entrance window of a 30 GHz VIPA (LightMachinery, OP-6721-3371-4) using a 300 mm cylindrical lens. The output of the VIPA passes through a 30 GHz etalon (LightMachinery, OP-7423-3371-2). The tilts of the VIPA and etalon are matched along the *y*-axis. Along the *x*-axis, however, the VIPA remains untitled while the etalon is tilted slightly such as to overlap the resonances of the VIPA and etalon. A 200 mm achromatic doublet imaging lens is used with a CMOS camera (PixeLINK, PL-D795MU) to capture the spectrally dispersed signal. The spectrometer and confocal scheme are depicted in Figs. 1(b) and 1(c). Setup parameters, performance metrics, and characterizations are reported in the Supplementary Material (Table S1, Fig. S1).

#### 2.2.4 Line-scanning scheme and 532 nm spectrometer

The line scanning scheme is depicted in Figs. 1(d) and 1(e). It uses a 532 nm diode-pumped solid-state laser (Coherent, Compass 315M-80, California, United States) that is first coupled to a SMF before being routed toward the objective using a fiber collimator. The beam is focused into the sample by an underfilled 10× objective. The incident power on the sample was set to 50 mW. For the contrast and diluted milk measurements, an etalon (LightMachinery, OP-7423-3371-1, Ontario, Canada) was inserted between the collimator and the objective and angled such that it transmits the main laser mode while blocking the side mode. Orthogonal to the optical axis of the laser, a pair of 50 mm lenses are used to approximately reimage the Rayleigh range of the focused laser through an XY adjustable slit. The latter is closed along the dispersion axis until the Brillouin signal is slightly reduced, and it is set to ∼110  μm along the orthogonal axis. This sets the field of view of the line measured to ∼110  μm. The light is then collimated using a 75 mm achromatic doublet and coupled via a 400 mm cylindrical lens into a 30 GHz VIPA (LightMachinery, OP-6721-3371-2) followed by a 30 GHz etalon (LightMachinery, OP-7423-3371-1). The tilts of both optics have similar relationships to those described above for the 785 nm spectrometer. A 300 mm achromatic doublet is used with a CMOS camera (Basler, daA3840-45um, Ahrensburg, Germany) to capture the spectrally dispersed signal. Setup parameters, performance metrics, and characterizations are reported in the Supplementary Material (Table S2, Fig. S2). Although we did not demonstrate it here, we note that the proposed cascade scheme is compatible with the dual line-scanning configuration developed to reduce artifacts associated with single-line illumination.^20^

#### 2.2.5 Sample preparation

All samples measured with the confocal setup were in glass cuvettes, whereas those measured with the line-scanning geometry were in polycarbonate or acrylic cuvettes. The porcine eyes were sourced from a local slaughterhouse and dissected within 4 h of death. The sclera was first incised, providing access to the vitreous chamber. The exposed vitreous humor was separated and placed into a cuvette [without phosphate buffered saline solution (PBS)]. With posterior access to the lens, the suspensory ligaments of the lens were cut, separating the lens from the ciliary body. The isolated lens was placed into a separate cuvette filled with PBS. Brillouin measurements were performed within 4 h of the dissection. The milk dilution consisted of 1 part of 3.25% milk diluted in 1000 parts of water by volume for the Brillouin measurements and ∼1 part of milk diluted in 30 parts of water for the contrast measurements.

#### 2.2.6 Spectral calibration, contrast characterization, and Brillouin measurements

The frequency calibration along the *y*-axis of the spectrometer sensor consisted of extracting the VIPA (and etalon) dispersion based on the observed spacing of successive orders and the known FSR. One to three orders were included to generate both the extinction and Brillouin spectra. To overcome the limited dynamic range of the camera, the extinction spectra were produced by combining multiple images taken at different exposures.

For all Brillouin spectra other than those used to characterize the precision and signal-to-noise ratio (SNR), the exposure was set to 250 ms for the confocal measurements and 500 ms for the line-scanning measurements. These were averaged by 2 to 16 images, depending on the signal strength. When characterizing the precision, 10 (confocal) or 15 (line-scanning) successive images were acquired for each exposure time, and a linear combination of two Lorentzian profiles (one of which is centered near zero) was fitted to the Stokes and anti-Stokes peaks to extract the Brillouin shift, the precision being taken as the standard deviation of the 10 or 15 shifts. The SNR is taken as the ratio of the averaged fitted Brillouin peak amplitude to its standard deviation.^25^

## 3 Results

### 3.1 Simulations

**Fig. 2:**
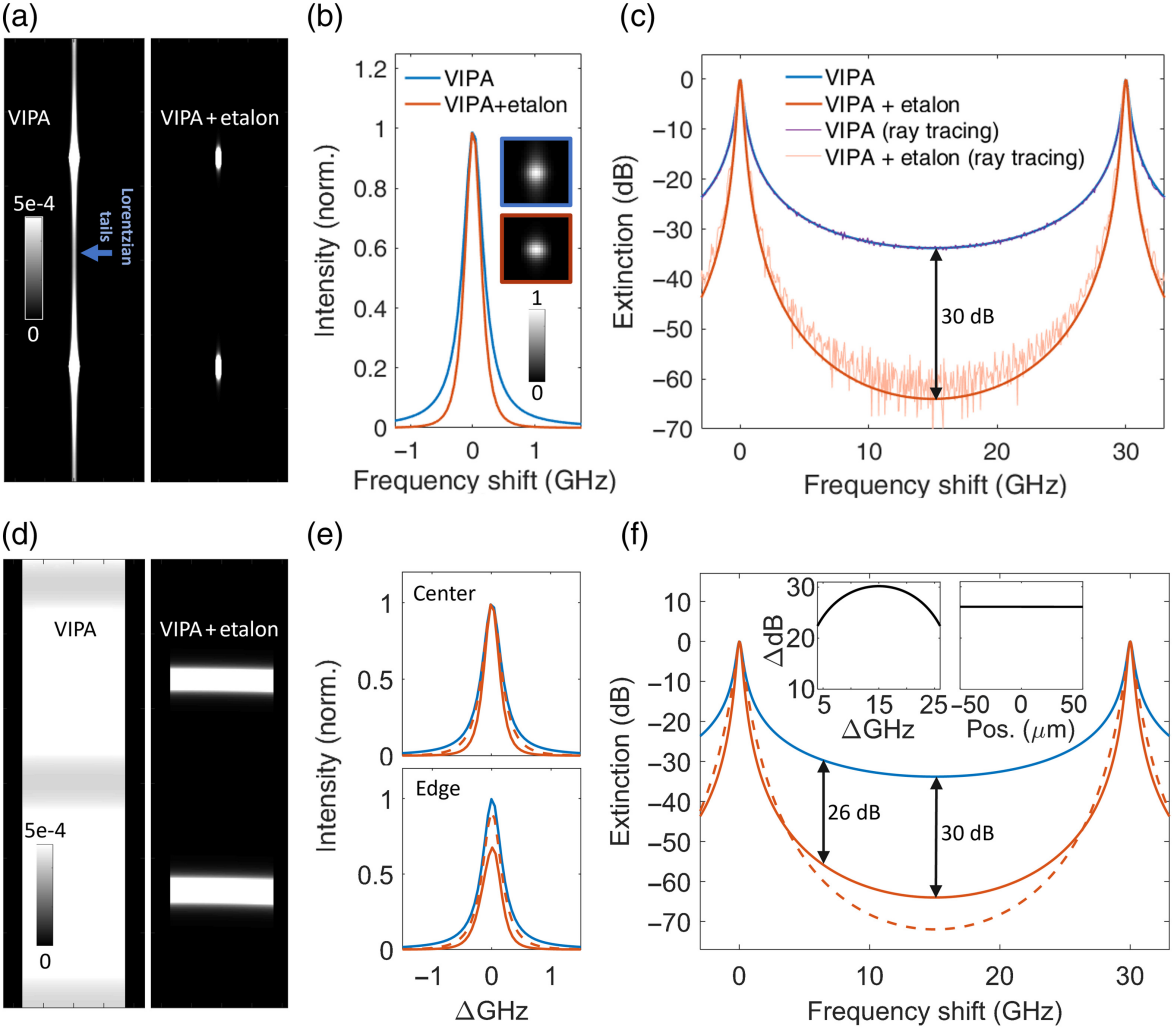
Simulated performances with perfect cavities and optimal alignment. (a) Saturated images of the sensor when an SLM laser is fiber-coupled to the VIPA spectrometer without (left) and with (right) cascading etalon. (b) Spectral profiles of one of the peaks. The insets show corresponding unsaturated sensor images. (c) Spectral profiles (dB scale) of the central VIPA FSR without and with the cascaded etalon. The result from ray-tracing simulations is also included. (d) Saturated images of the sensor with line-scan illumination over 110  μm, assuming only Rayleigh scattering, for the VIPA-only and VIPA-etalon cases. (e) Spectral profile of one order, in the center and on the right edge of the line source. (f) Spectral profiles (dB scale) in the center of the line source. The left and right inset figures show the contrast enhancement as a function of the frequency shift and position along the line (for the 5-8 GHz window), respectively. For both (a) and (d), the color scale is shared by both panels and refers to an otherwise normalized image. The legend for (e, f) is the same as that shown in (b), except for the dashed red line, which corresponds to the case of two cascaded low-finesse etalons.

The effect on spectral contrast and throughput when cascading a VIPA with a matched etalon was modeled, assuming ideal theoretical performance and considering the signal from an SLM laser. Suitable optical configurations were analyzed for confocal geometry in Figs. 2(a)–2(c) and for line-scanning geometry in Figs. 2(d)–2(e). Figure 2(a) compares the simulated sensor image for the VIPA-only case and VIPA-etalon case, demonstrating the etalon’s suppression of the light between resonance orders. Assuming defect-free cavities and optimal alignment, the transmission achieves unity at resonance. This is seen in Fig. 2(b), which also highlights the significant suppression of the Lorentzian tails. It is also worth noting that the full width at half maximum (FWHM) of the instrument response function is slightly reduced in the VIPA-etalon case, as expected when the etalon’s finesse is nonnegligible compared with that of the VIPA. Figure 2(c) displays the modeled extinction curve without and with the cascaded etalon, revealing a 30 dB increase in spectral contrast in the central region. The intrinsic ∼33  dB contrast for the VIPA-only case aligns with previously reported values.^9^^,^^14^ Complementary ray-tracing simulations provided further validation, showing good agreement except for a slightly reduced contrast due to lens aberrations and other practical limitations. Although 30 GHz FSR cavities were used in this study, smaller FSRs are better suited for most biological samples to optimize performance, particularly with red or infrared excitation.

Along the direction orthogonal to the VIPA dispersion axis, the spectrometer input is re-imaged onto the image sensor with a magnification determined by the selected optics. Consequently, the confocal (fiber-coupled) geometry produces a series of dots, whereas the line-scanning geometry generates a series of lines. This characteristic pattern is observed in Fig. 2(d), which demonstrates the cascade concept for line-scanning geometries (or MMF inputs). Similarly to the confocal case, the light between resonance orders is suppressed by the cascaded etalon. Figure 2(e) displays the normalized profiles in the middle and on the edge of the line, showing a reduced FWHM and suppressed tails for the VIPA-etalon case. Although the line is not globally collimated, the light from each individual point along the line is nominally collimated (but has a different incident angle on the VIPA-etalon pair), such that the etalon transmission is relatively high along the entire line. However, the transmission is lower on the edges. This can be understood from the consideration that the VIPA-etalon case involves overlapping two sets of fringes. The midpoints of each set of fringes are consistent in their vertical position across both elements, but the *x*-tilt of the etalons—required to ensure that no back reflections overlap the sensor’s region of interest—slightly inclines its fringes relative to those of the VIPA (see Fig. S3 in the Supplementary Material). This inclination limits the region along the line axis, where fringe overlap can be achieved, particularly as the required *x*-tilt increases with the target length, amplifying this effect. As higher etalon finesse imposes stricter overlap constraints, exacerbating this limitation and reducing peak transmission, multiple etalons with lower finesse are preferred over a single high-finesse etalon, especially in line-scanning geometries. For comparison, Fig. 2(e) also displays the profiles obtained with two cascaded etalons of finesse 11, showing higher transmission on the edge. The extinction curves in Fig. 2(f) corresponding to a single high-finesse cascaded etalon and two low-finesse cascaded etalons show contrast enhancements of 30 dB (26 dB on average in the 5 to 8 GHz range) and 34 dB, respectively.

**Fig. 3:**
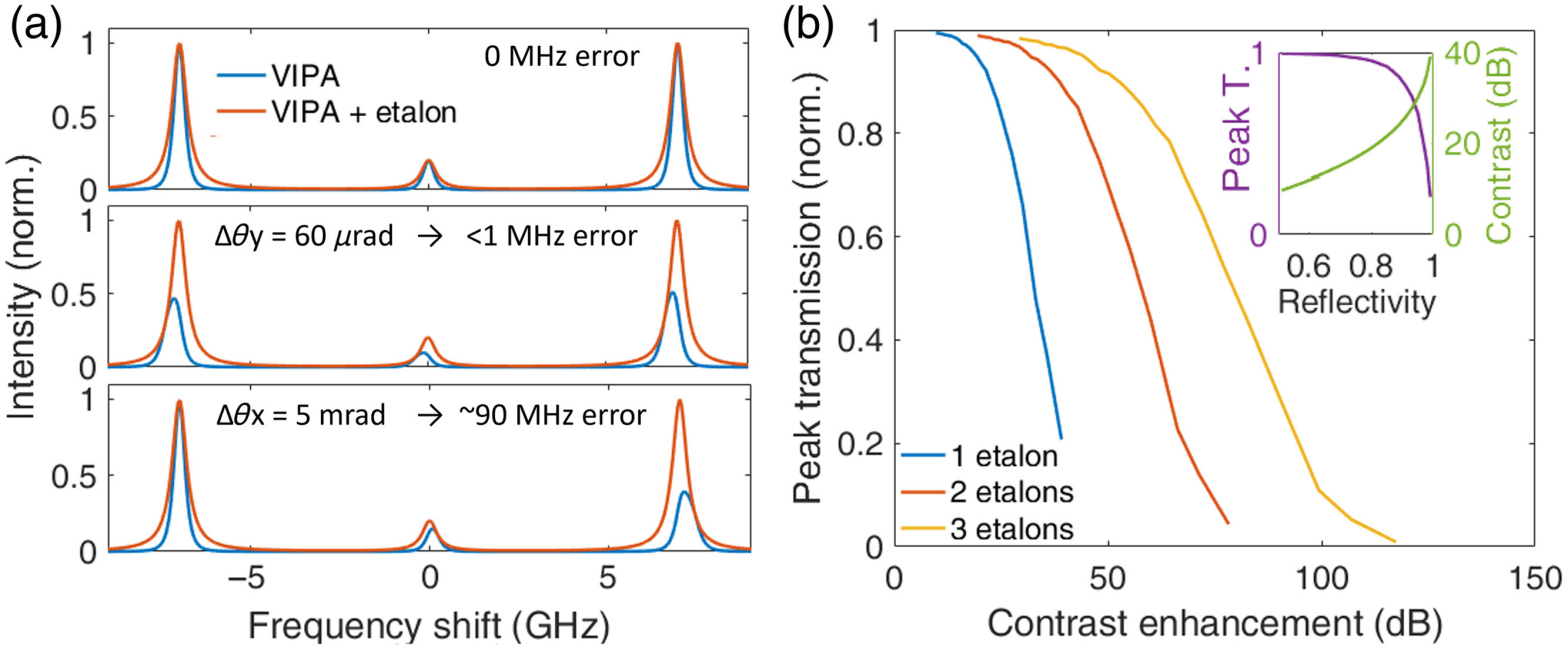
Simulated impact of imperfect alignment and cavity defects. (a) VIPA-etalon Brillouin spectra with optimal alignment (upper), slight mismatch in y-tilt (middle), and slight mismatch in *x*-tilt (bottom). Note that the *x*-tilt mismatch of 5 mrad requires a *y*-tilt adjustment of 1 mrad to retrieve the overlap for one of the Brillouin peaks. The red curves are VIPA-only, whereas the blue curves are the VIPA-etalon. (b) Peak transmission as a function of the contrast enhancement for one to three cascaded etalon stages. The inset shows the peak transmission and contrast enhancement as a function of the etalon’s surface reflectance (one stage).

With optimal alignment, as assumed in Fig. 2, the retrieved Brillouin shift is unaffected by the presence of the cascade. This is shown in the upper panel of Fig. 3(a), which simulates a Brillouin signal with a 7 GHz shift. Interestingly, if there is a slight *y*-tilt mismatch between the VIPA, and the etalon while the effective thickness remains matched, the peak transmission is reduced, but the recovered Brillouin shift remains unaffected, as depicted in the middle panel of Fig. 3(a). Consequently, the reduced transmission at the edges of line sources does not introduce significant accuracy errors in the retrieved Brillouin shift. It also renders the cascade configuration robust against slight alignment drifts. However, if there is a mismatch in the effective thickness of the VIPA, and etalons (e.g., if the *x*-tilt or temperature tuning fails to compensate for the actual thickness mismatch), the cascade affects both throughput and the spectral separation between the Brillouin peaks, as shown in the lower panel. Although this could be calibrated, ensuring an effective thickness match is preferable. Here, too, alignment requirements are less stringent with low-finesse etalons.

Although perfect cavities were assumed in Fig. 2, real-world defects limit the finesse, contrast, and peak transmission of both VIPAs and etalons (though near-unity throughput is preserved in the former case). The impact of thickness nonuniformity can be estimated by generating a normal distribution of thicknesses centered on the nominal thickness and averaging their spectral responses. The inset of Fig. 3(b) shows the resulting peak transmission and contrast for a single etalon with a thickness nonuniformity of 1.5 nm (standard deviation), typical for etalons.^26^ The main figure illustrates the achievable peak transmission for single-, double-, or triple-stage cascades as a function of target contrast enhancements, further highlighting the advantage of multiple low-finesse etalons over a single high-finesse etalon.

Another aspect that is trivially dealt with in simulations but that can complicate practical implementation is in thickness matching. In practice, unless great efforts are made, the fabricated etalons and VIPA will vary somewhat in thickness. In fact, given that a nonzero *x*-tilt is required to prevent back-reflection issues, as further described in the next section, a very small but non-zero thickness mismatch is ideal. In principle, a large range of thickness mismatch can be compensated for in confocal geometry by adjusting the *x*-tilt. In practice, we find the performance to degrade if the mismatch is such that the required *x*-tilt is large. The constraint is greater in line-scanning geometry, for which optimal performance requires a specific effective thickness mismatch. If problematic, this can be addressed with a temperature-controlled mount, but this was not necessary for this study.

### 3.2 Experimental Characterization and Demonstration in Confocal Geometry

**Fig. 4:**
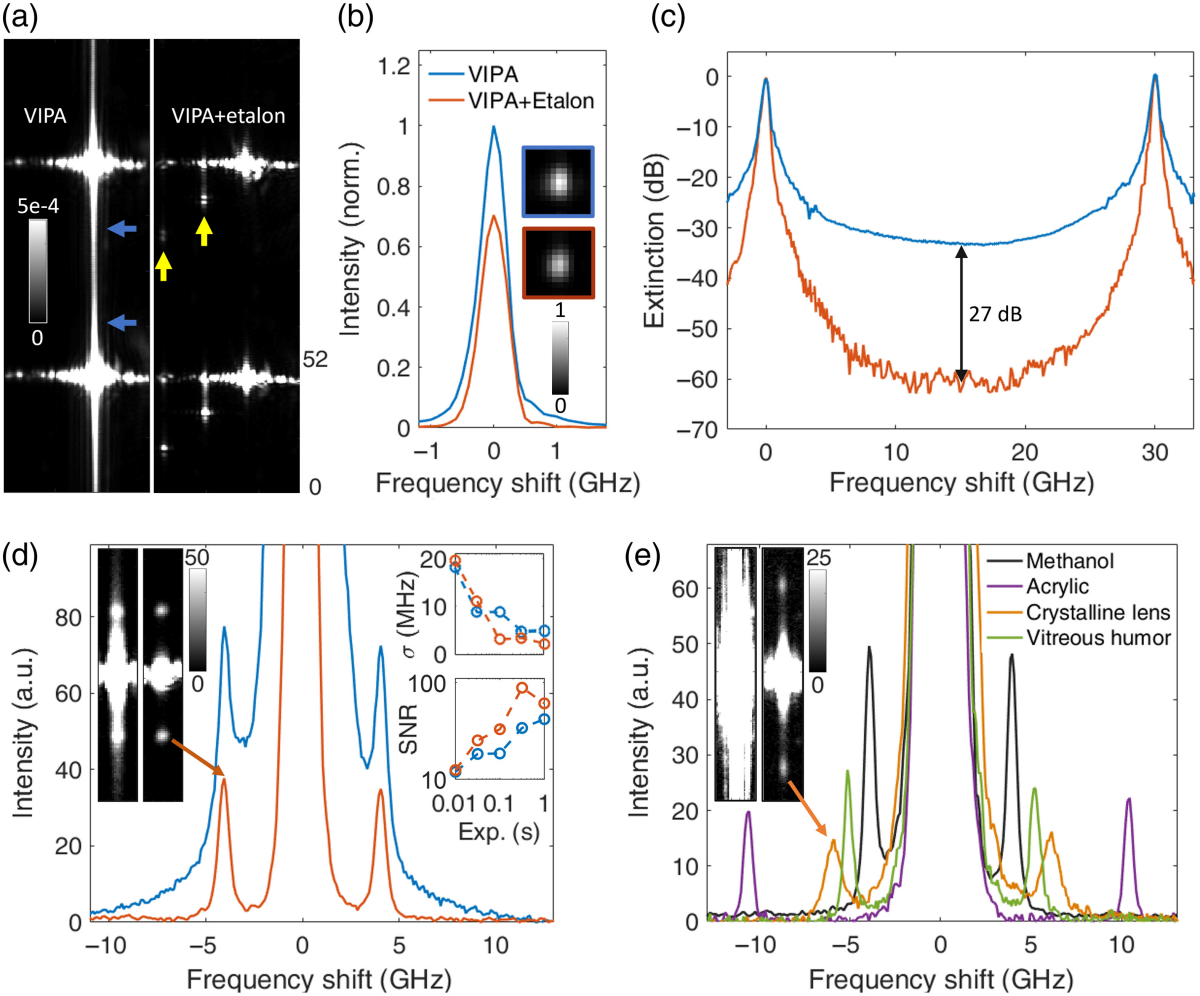
Experimental assessment in confocal geometry. (a) Saturated images of the sensor when the 785 nm laser is fiber-coupled to the VIPA spectrometer without (left) and with (right) cascading etalon. The color scale is shared by both panels and refers to an otherwise normalized intensity scale (both cases were normalized individually, and the exposure was increased accordingly to access the 0 to 5e-4 (i.e., 0% to 0.05%) relative range). The blue and yellow arrows identify Lorentzian tails and back reflection artifacts, respectively. (b) Spectral profiles of one resonance peak, normalized to the VIPA-only case. The insets show the corresponding sensor images. The same legend applies to (b)–(d). (c) Spectral profiles (dB scale) of the central FSR of the VIPA without and with cascaded etalon. (d) Brillouin spectrum of ethanol acquired without and with cascade. The left inset shows the corresponding sensor images for one of the orders. The upper and lower right insets show the precision of the Brillouin shift and SNR, respectively, as a function of the exposure time. (e) Brillouin spectra of various other samples obtained with the VIPA-etalon cascade. The inset shows sensor images of the porcine lens without and with cascaded etalon.

The experimental verification of the VIPA-etalon cascade configuration for a fiber-coupled confocal setup is presented in Fig. 4. Figure 4(a) shows the sensor images obtained when coupling the 785 nm SLM laser to the spectrometer, both without and with the cascaded etalon, confirming the suppression of the light between VIPA orders. The behavior closely matches the modeled results in Fig. 2(a) along the axis of interest, i.e., the VIPA dispersion axis. Horizontally, additional features due to aberrations and scatter are observed in highly saturated experimental images for both the VIPA-only and VIPA-etalon cases; however, these have no consequences for the retrieved VIPA spectra. Of greater interest are the additional features unique to the VIPA-etalon case, corresponding to back reflections from the cascaded etalon that reach the sensor after one or multiple round trips between the two optics. As noted above, a non-zero *x*-tilt (and thus a slightly mismatched physical thickness or temperature) is required to prevent these reflections from overlapping with the Brillouin signals located between VIPA orders. The spectral profile of one interference order, shown on a linear scale in Fig. 4(b), indicates that the cascaded etalon does not distort the spectral response, has negligible impact on the FWHM (∼0.51  GHz in both cases), and reduces peak transmission by only ∼30%. These outcomes are consistent with imperfect etalons of moderate finesse cascaded with a high-finesse VIPA. Finally, the extinction curve in Fig. 4(c) closely resembles its modeled counterpart, demonstrating a 27 dB (∼500 fold) contrast enhancement beyond the ∼33  dB contrast of the VIPA alone, achieving a total contrast slightly exceeding 60 dB.

The performance of the VIPA-etalon cascade was further demonstrated and studied in the context of Brillouin measurements. Figure 4(d) compares the measured spectrum of ethanol without and with the cascaded etalon. As ethanol, a transparent sample, can also be measured with the VIPA alone, it provides a basis for assessing the cascade’s impact on the accuracy, precision, and SNR of the retrieved Brillouin shift. By fitting a linear combination of two Lorentzian profiles to account for the tail of the elastic peak in the VIPA-only case, the extracted Brillouin shift from the VIPA-only and VIPA-etalon spectra differed by 2 MHz. The extracted average FWHM was 0.73 GHz for the VIPA-only case and 0.64 GHz for the VIPA-etalon case; the smaller FWHM of the latter is consistent with the reduced tails of its instrument response function. Interestingly, the precision was similar in both cases, as shown in the inset, despite the reduced peak transmission with the cascaded etalon. Furthermore, the achieved precision is comparable to the suggested benchmark when adjusting for wavelength, sample, power, and quantum efficiency.^27^ Consistent with reduced tails and thus diminished sensitivity to Rayleigh peak fluctuations, despite reduced peak transmission, the SNR was greater in average for the VIPA-etalon configuration, as shown in the inset. We note that camera noise is non-negligible and the spectrometer does not operate in a shot-noise-limited regime. Additional reference samples measured with the VIPA-etalon cascade are displayed in Fig. 4(e). As shown in the figure inset, the tails of the elastic scattering peak compromised the ability of the VIPA-only spectrometer to measure the Brillouin signal from the porcine crystalline lens under our experimental conditions. In contrast, the VIPA-etalon enabled clear detection of the signal. Finally, to assess the stability of the cascade scheme, we tracked the Brillouin shift of an acrylic sample over 4 h without performing adjustments [see Fig. S6(a) in the Supplementary Material]. Despite using low-cost mounts and lasers and no vibration isolation, we observed a standard deviation of only 7.2 MHz.

### 3.3 Experimental Demonstration in Line-Scanning Geometry

**Fig. 5:**
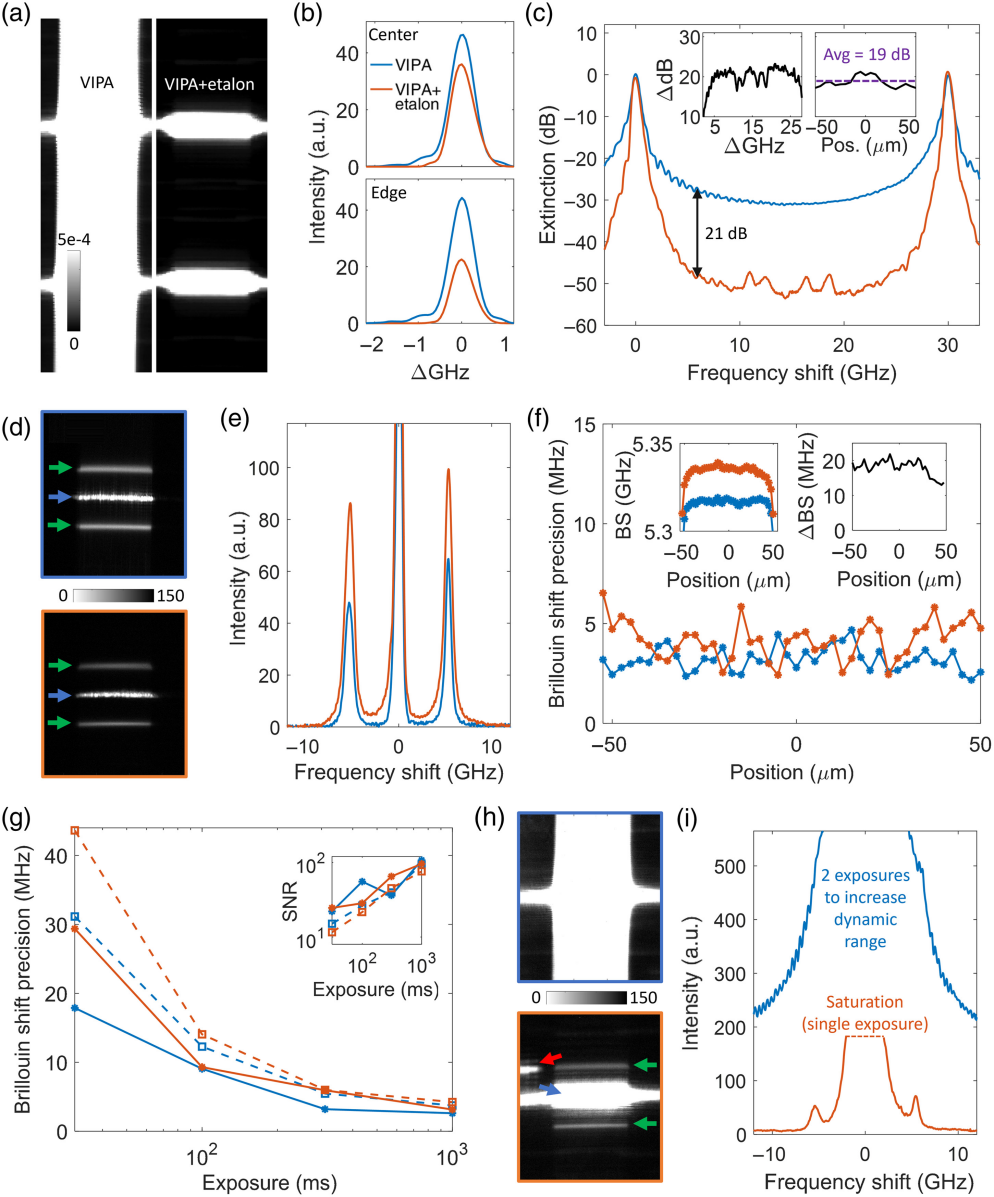
Experimental assessment in line-scanning geometry. (a) Saturated images of Rayleigh signals without (left) and with (right) cascading etalon. The color scale is shared by both panels and refers to an otherwise normalized intensity scale (both cases were normalized individually, and the exposure was increased accordingly to access the 0 to 5e-4 (i.e., 0% to 0.05%) relative range). (b) Spectral profiles of one resonance peak, normalized to the VIPA-only case, in the center (upper panel, averaged over 50  μm) and edge (lower panel, averaged over 25  μm) of the line. The same legend applies to (c, e, f, g, i). (c) Spectral profiles (dB scale) of the central FSR of the VIPA without and with cascaded etalon, in the center (averaged over 10  μm) of the line. The left and right insets show the contrast enhancement as a function of the frequency shift (in the center of the line) and position along the line (averaged over the 5 to 8 and 22 to 25 GHz windows), respectively. (d) Sensor images of the Brillouin signal from water without (upper) vs. with (lower) cascaded etalon. The blue and green arrows identify the Rayleigh and Brillouin signals, respectively. (e) Corresponding Brillouin spectra in the center of the line-scan. (f) Precision of the water Brillouin shift as a function of the position along the line without and with cascaded etalon (500 ms exposure). The left and right insets show the Brillouin shift as a function of the position along the line for both cases and their difference, respectively. (g) Precision of the water Brillouin shift as a function of the exposure time. The inset shows the corresponding SNR as a function of the exposure time. The solid lines result from averaging three orders to obtain the Brillouin spectra (as was used for (e, f, g, i)), whereas the dashed lines consider only the central order. (h) Same as (d) but for the milk dilution. The red arrow identifies an artifact from the cascade etalon. (i) Corresponding Brillouin spectra in the center of the line-scan. The spectrum for the VIPA-only case was obtained by combining a second lower exposure because it was almost entirely saturated at 500 ms.

The proposed VIPA-etalon cascade configuration was experimentally validated using a line-scanning collection geometry. Due to limited parts availability, a single high-finesse etalon cascade stage was used instead of the superior two-stage configuration alternative included as a dashed line in Figs. 2(e) and 2(f). Figure 5(a) shows saturated sensor images obtained without and with the cascaded etalon when focusing the excitation laser into a highly turbid sample (chosen to produce a strong Rayleigh signal and negligible Brillouin signals in comparison). Similarly to the modeled results shown in Fig. 2(d), the light between VIPA orders is suppressed by the cascaded etalon. The spectral profiles of one interference order, in the central region and near the edge of the line-scan, are shown on a linear scale in Fig. 5(b). Consistent with the modeled profiles shown in Fig. 2(e), the cascaded etalon suppresses the tails, reduces slightly the instrument response’s FWHM (from 0.65 to 0.63 GHz in the center and from 0.65 to 0.59 GHz on the edge), and reduces the transmission on the edge of the line compared to the center. Also observed experimentally is a ∼25% reduction in peak magnitude for the central profile and a slight asymmetry introduced by the etalon. Finally, the extinction curve shown in Fig. 5(c) is qualitatively similar to its modeled counterpart shown in Fig. 2(f) but has two important differences: the overall contrast improvement provided by the cascade etalon is lower, and there are artifacts in the 10 to 20 GHz region. A major culprit was found to be pixel diffraction artifacts of the camera itself, as shown in Fig. S4 in the Supplementary Material. The contrast improvement achieved in the 5-8 GHz region, averaged over the length of the line, was 19 dB (∼80 fold), in comparison to the modeled value of 26 dB.

Sensor images from a water sample, obtained without and with the cascaded etalon, are presented in Fig. 5(d). The pattern remained unaffected by the cascaded etalon other than having reduced intensity and slightly narrower spectral features. The corresponding Brillouin spectra at the central position, reconstructed from three orders, are shown in Fig. 5(e), revealing an approximate 40% reduction in peak transmission. The average FWHMs of the Brillouin peaks were 1.04 and 0.89 GHz for the VIPA-only and VIPA-etalon setup, respectively. Figures 5(f) and 5(g) show that the precision and SNR of the Brillouin shift for the VIPA-only and VIPA-etalon cases are similar in the middle of the line (with the VIPA-only case having only a slight advantage), but the inclusion of the cascaded etalon reduced the precision near the edges. A small reduction in precision for transparent samples producing minimal Rayleigh scattering tails is expected for any contrast-enhancing scheme with nonzero transmission loss. We note that the spectrometer does not operate in a fully shot-noise-limited regime. The cascaded etalon caused a slight offset (averaged at 18 MHz along the line) to the Brillouin shift; this could be compensated for during the calibration or prevented by achieving better effective thickness matching. The VIPA-etalon cascade performance was further demonstrated with a turbid sample (diluted milk). Sensor images in Fig. 5(h) show that, in the VIPA-only case, the elastic peak tails, which are orders of magnitude larger, completely concealed the Brillouin signals. Upon cascading the etalon, the Brillouin peaks were uncovered, enabling the extraction of a clear spectrum, as shown in Fig. 5(i). In addition, the lower panel in Fig. 5(h) (see also Fig. S5 in the Supplementary Material) reveals an echo of the main orders, like those of the right panel of Fig. 4(a), caused by back reflections from the cascaded etalon reaching the sensor. Care must be taken to ensure these reflections do not overlap with the sensor regions capturing the Brillouin signal using a sufficiently large *x*-tilt for the etalon. It is also advantageous to increase the distance between the etalon and VIPA in the line-scanning geometry to minimize them (we spaced the VIPA and etalon by “∼2” in confocal geometry and “∼5” in line-scanning geometry). Finally, we assessed the stability of the cascade scheme in line-scanning geometry by tracking the Brillouin shift of water over 4 h without performing adjustments and observed a standard deviation of only 5.6 MHz [see Figs. S6(b) and S6(c) in the Supplementary Material].

## 4 Discussion

The addition of cascaded etalon stages is an inexpensive way to drastically augment the performance of VIPA spectrometers for both point and line sources without increasing the size and complexity significantly. In principle, a very large contrast enhancement with minimal penalty on throughput could be achieved given optimal etalons, although in practice, employing low-cost, off-the-shelf etalons and cameras reduces achievable performance. This configuration has various advantages and tradeoffs with previously reported contrast enhancement methods. The commonly employed multistage crossed VIPA scheme enhances the contrast by ∼25–30  dB in its standard double-stage implementation and by ∼50  dB in triple-stage or upon combining a double stage with another technique such as coronagraphy.^9^^,^^17^^,^^27^ Compared with the proposed VIPA-etalon cascade scheme, multistage crossed VIPA configurations have less strict alignment requirements and no constraints regarding thickness matching, but they are more costly, bulky, and are not compatible with line-scanning. Cross-dispersing a VIPA with an echelle enhances the contrast by ∼25  dB in confocal geometry^28^ but is not amenable to additional stages, and the enhancement is mostly eliminated when using line sources. Background deflection with a rhomboidal-shaped mask is a relatively simple and inexpensive method that has been shown to enhance contrast by ∼40  dB,^18^ but multistage implementation and compatibility with line-scanning are unlikely. Intensity-equalization methods based on apodization filters^29^ or spatial light modulators^14^ are compatible with line-scanning geometries, but the demonstrated contrast enhancement of apodization filters has been limited to 15 to 20 dB, and spatial light modulators are costly and have only been demonstrated with confocal geometries. Another approach previously reported for confocal geometry and relevant to our work consists of inserting a low-finesse etalon in a multipass configuration within the collimated beam upstream of a two-stage VIPA spectrometer.^30^ The etalon acts as a narrow bandpass filter, with ∼3  GHz bandwidth in the authors’ case, angle-tuned to transmit the anti-Stokes peak while rejecting the Rayleigh peak and Stokes peak. This setup leads to a restriction to a narrow spectral window, which contrasts with the broad coverage on both sides of the Rayleigh peak desired for versatile measurements and maintained by most techniques discussed in this section, including our downstream cascade approach, which relies on angular presorting by the VIPA.

We note that the insertion loss we obtained with the cascade etalon (on the order of 2 dB over 2 to 3 VIPA orders) is comparable to most methods noted above. Insertion losses increase the exposure time required to achieve a target precision or SNR. When the elastic scattering signal is relatively small (e.g., a transparent sample measured in line-scanning geometry), a nonzero insertion loss unavoidably results in reduced precision and SNR (for a given exposure time) compared to the single-stage VIPA reference, independent of the contrast enhancement. However, this is not common for biological applications. In the typical case where the elastic scattering signal is overwhelmingly large (e.g., most biological samples in both confocal and line-scanning geometries, especially near interfaces), contrast enhancement largely compensates for a small insertion loss, resulting in precision and SNR that are higher than those obtained with a single-stage VIPA or, very often, simply makes the detection of Brillouin peaks possible when it would otherwise be impossible. It remains desirable in any case to minimize the insertion loss of contrast enhancement (and Rayleigh suppression) schemes, and in our case, it could be reduced by employing two or more stages of lower finesse etalons, as shown in Fig. 3.

Finally, the VIPA-etalon cascade configuration also has various advantages compared with rubidium cells, one of the highest-performing Brillouin notch filters and the primary approach employed to circumvent VIPA contrast limitations in line-scanning geometry.^20^^,^^22^^,^^31^ Rubidium cells impose the use of a frequency locked laser and require significant heating, which adds cost and complexity. Moreover, it imposes the use of 780 nm excitation, which is suboptimal for certain samples due to the $\lambda^{-4}$ dependence of Brillouin signal intensity^32^ and the reduced quantum efficiency of silicon sensors in the near infrared. Iodine cells can be used for 532 nm excitation, but a cluster of bands complicates Brillouin measurements.^16^ On the other hand, gas cells have the advantages of not imposing limitations on the line source length, being essentially alignment-free, achieving a greater effective contrast enhancement per stage (∼40  dB), and limiting potentially detrimental artifacts from reaching the sensor by suppressing the elastic peak rather than directly enhancing the spectrometer contrast. The optimal approach varies by context and application, requiring user evaluation to select the most suitable method.

## 5 Conclusion

We developed a novel Brillouin spectrometer scheme based on a VIPA-etalon cascade and demonstrated, through computational simulations and experimental characterization, that this configuration enhances contrast by orders of magnitude with minimal throughput loss. Experimentally, we achieved a contrast enhancement of 27 dB (∼500-fold) in confocal geometry and ∼19  dB (∼80-fold) in line-scanning geometry, enabling measurements of turbid samples inaccessible with single-stage VIPA spectrometers while minimally affecting precision, speed, and accuracy for transparent samples. Future enhancements include implementing multiple lower finesse cascaded etalons for improved performance. This simple, compact, and cost-effective scheme, compatible with various collection geometries and across a wide range of wavelengths, can be easily implemented in existing setups, facilitating new biomedical applications.

## Supplementary Material

10.1117/1.JBO.31.2.026001.s01

## Data Availability

The data presented in this article are publicly available in Zenodo at https://doi.org/10.5281/zenodo.16945684 The archived version of the code described in this manuscript can be freely accessed through GitHub at https://github.com/HubertJeanRuel/VIPAEtalonCascade
