# Supplementary material for: High-performance Brillouin spectroscopy using VIPA-etalon cascades
Source: J Biomed Opt. 2026 Feb 3;31(2):026001. doi: 10.1117/1.JBO.31.2.026001 (PMC12867489; doi:10.1117/1.JBO.31.2.026001)
Supplement: Supplementary file 1 [file JBO_031_026001_SD001.pdf]

Supplementary Material

High-Performance Brillouin Spectroscopy using VIPA-Etalon Cascades

Sophie Chagnon-Lessard[a], Julian Nicolai[a], Joshua Steller[a], Eng Kuan Moo[b], Hubert Jean-Ruel[a,*]
[a] Carleton University, Faculty of Engineering and Design, Department of Electronics, Ottawa, Canada
[b] Carleton University, Faculty of Engineering and Design, Department of Mechanical and Aerospace Engineering, Ottawa, Canada

Table S1. Reporting table for the confocal collection modality (inspired by Bouvet *et al.* [33]).

	**VIPA-only**	**VIPA-etalon**	**Note**
System	Single-stage VIPA	Single-stage VIPA with cascaded etalon	
Excitation objective lens NA	0.25 NA, underfilled		The $1/e^2$ diameter of the incident beam is ~ 2 mm.
Illumination wavelength	785 nm		-
Illumination type	Point		-
Detector type	cMOS		-
Scattering angle	180°		-
Polarization probed/analyzed	Circular		-
X-optical resolution	~8 μm		See Fig. S1(a)
Y-optical resolution	~8 μm		See Fig. S1(a)
Z-optical resolution	~58 μm		See Fig. S1(b)
Spectral resolution	0.51 GHz	0.51 GHz	See Fig. 4(b)
Unambiguous spectral range	-15 to 15 GHz		30 GHz FSR VIPA (and etalon)
Average laser power on sample	30 mW		-
Single spectrum acquisition time (used for metrics below)	100 ms		-
Brillouin frequency shift precision	9 MHz	3 MHz	See Fig. 4(d)
Signal-to-noise ratio	18	33	See Fig. 4(d)

Table S2. Reporting table for the line-scanning collection modality.

	**VIPA-only**	**VIPA-etalon**	**Note**
System	Single-stage VIPA	Single-stage VIPA with cascaded etalon	
Excitation objective lens NA	0.25 NA, underfilled		The $1/e^2$ diameter of the incident beam is ~ 2 mm.
Collection lens NA	~0.13 NA		-
Illumination wavelength	532 nm		-
Illumination type	Line		-
Detector type	cMOS		-
Scattering angle	90°		-
Polarization probed/analyzed	Linear, unknown angle		-
X-optical resolution	<7 µm		See Fig. S2(e)
Y-optical resolution	~4 µm		See Fig. S2(c)
Z-optical resolution	<7 µm		See Fig. S2(e)
Spectral resolution	~0.65 GHz	~0.61 GHz	In average, see Fig. 5(b)
Unambiguous spectral range	-15 to 15 GHz		30 GHz FSR VIPA (and etalon)
Average laser power on sample	50 mW		-
Single spectrum acquisition time (used for metrics below)	1000 ms		-
Brillouin frequency shift precision	<5 MHz	<5 MHz	See Fig. 5(g)
Signal-to-noise ratio	107	96	See Fig. 5(g)

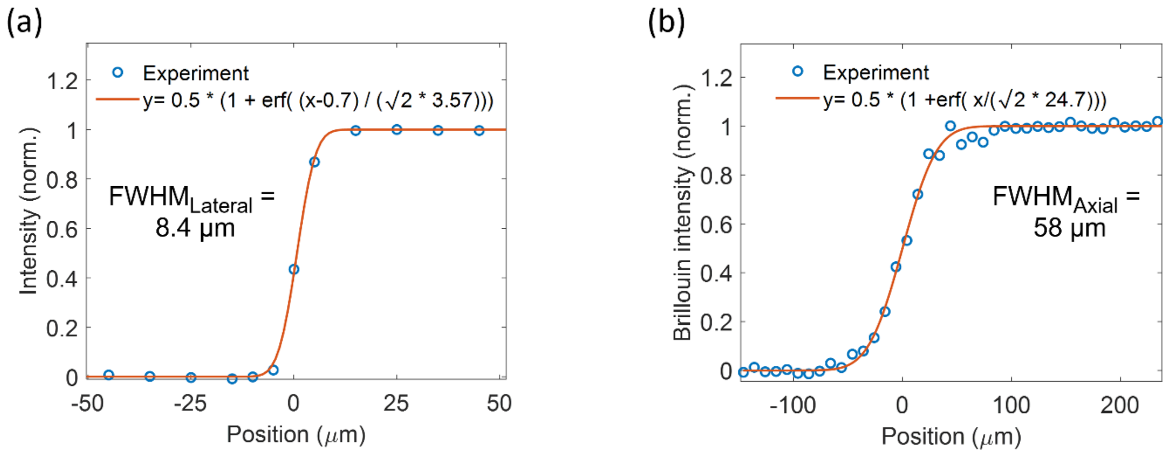

Fig. S1. Characterization of the spatial resolution for the confocal geometry. (a) Knife-edge measurement of the excitation beam at the focal volume. The error function fit yields a sigma parameter σ = 3.57 µm, corresponding to a FWHM of 8.4 µm. This value provides an estimate for the lateral resolution. (b) Raise of the Brillouin signal as an acrylic sample is translated axially from out-of-focus to in-focus. The error function fit yields a sigma parameter σ = 24.7 µm, corresponding to a FWHM of 58 µm. This value provides an estimate for the axial resolution. The spatial resolution is the same for both the VIPA-only and VIPA-etalon cases.

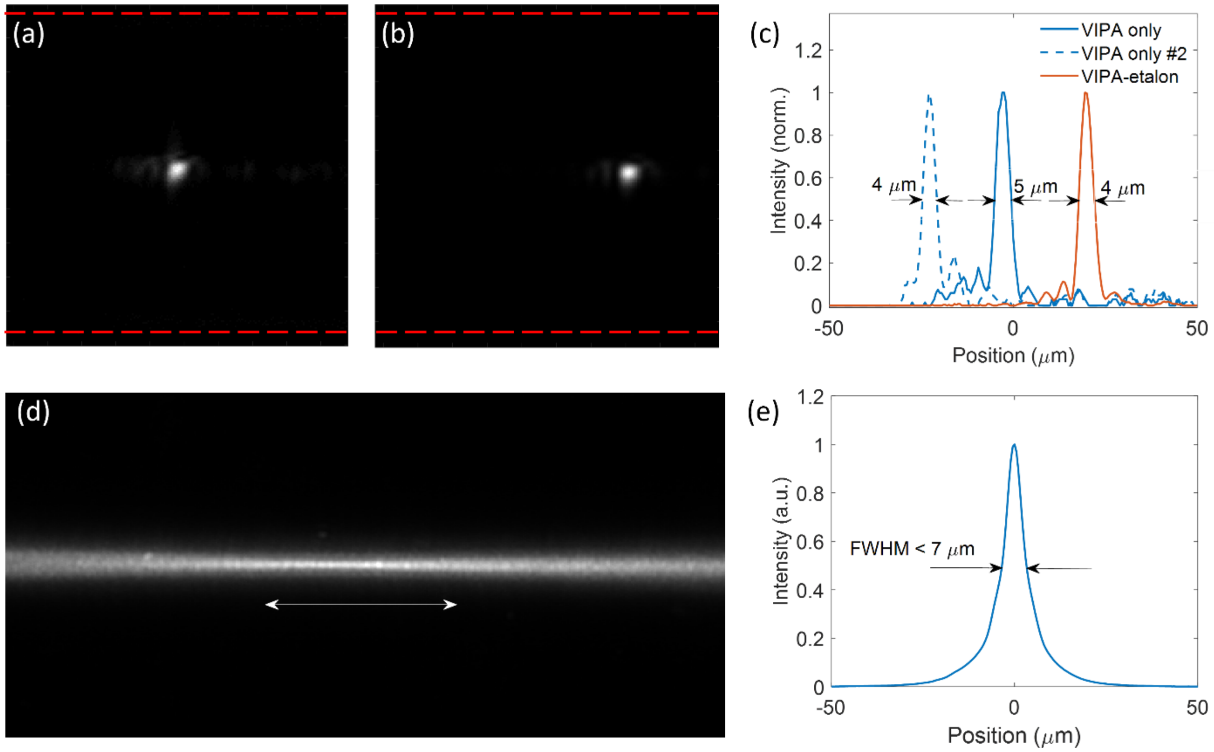

Fig. S2. Characterization of the spatial resolution for the line-scanning geometry. (a,b) Sensor image (covering a width of 100 µm in the sample, slightly narrower than the re-imaged line-scan width of 110 µm) showing a strong Rayleigh signal at the position along the line where an impurity passes by in the illuminated volume; the VIPA-only case is shown in (a) and the VIPA-etalon case in (b). The dashed red lines show the approximate position of the Brillouin signals (invisible at the employed exposure) for reference. (c) Profiles of the Rayleigh signals shown in (a,b) along the spatial axis (i.e. along the line axis, corresponding to the horizontal axis). An additional profile is shown for the VIPA-only case. The profiles' FWHM provide an approximate upper limit for the spatial resolution along y (referring to Fig. 1(d)'s axes). (d) Image of the laser focused in silicon fluid (DOWSIL™ 705 Diffusion Pump Fluid) acquired directly in the line-scanning setup after removing the cylindrical lens, VIPA, and etalon. The arrow is 200 pixels long, which corresponds to a length of 100 µm in the sample (as determined by imaging a ruler and consistent with 1:1 imaging of the excitation line onto the slit (via two lenses of 50 mm focal length) and subsequent 1:4 re-imaging on the sensor (via a 75 mm collimator and a 300 mm imaging lens)). (e) Cross-section (i.e. vertical profile) of the excitation line image, averaged over the central 100 µm. The profile's FWHM provides an approximate upper limit for the spatial resolution along x and z. The actual resolution is expected to be slightly better, particularly along x, because of the confocal slit (and the VIPA which acts itself as an additional confocal slit). We note that the spatial resolution estimates are the same for the VIPA-only and VIPA-etalon cases.

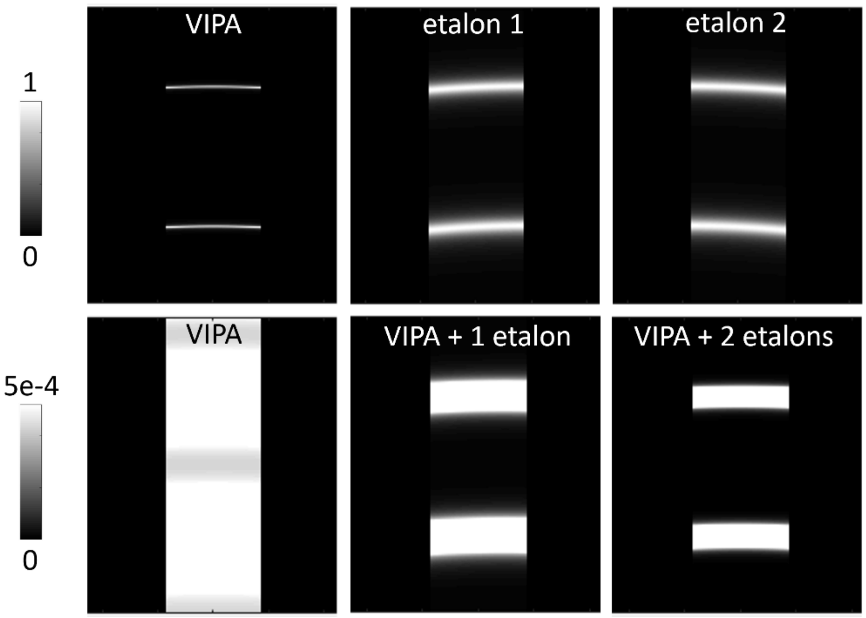

Fig. S3. Simulated sensor images for the line-scan geometry and dual-stage cascade case, assuming only Rayleigh scattering. The line's length was set to 150 µm. The upper row illustrates the normalized transfer function for each FP in isolation. The bottom row, from left to right, shows saturated images for the VIPA-only, VIPA-etalon, and VIPA-etalon-etalon cases (saturated to 2000 times the dynamic range).

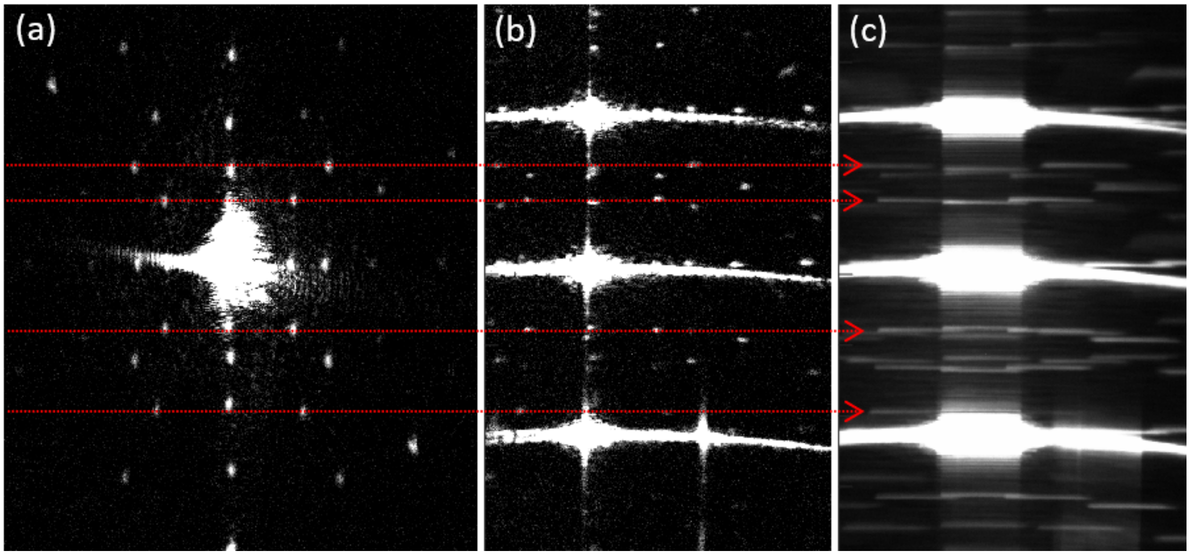

Fig. S4. Pixel diffraction artefacts limiting the measured line-scan contrast. (a) Saturated sensor image with a single point is illuminated on the camera (the cylindrical lens, VIPA, and etalon were not in the setup when this image was acquired). (b) Saturated sensor image with a single point significantly scattering along the line of the line-scan setup (with the cylindrical lens, VIPA, and etalon re-inserted in the setup). (c) Saturated sensor image with the entire line significantly scattering. A1:30 water:3.25% milk mixture was employed to generate (a-c). (a-b) were obtained by capturing the image when an impurity passed by the excitation volume. The multiple line artefacts observed in (c) and point artefacts in (b) are not caused by the cascaded etalon, but rather by camera pixel diffraction, as can be seen by the correspondence of their location with (a). The magnitude and location of the camera pixel diffraction artefacts vary with wavelength and from one camera to the other. They are a non-issue in confocal geometry since a slight rotation of the camera can prevent them from overlapping with the Brillouin signals. and following their locations along the red dotted lines. They are not caused by the addition of cascade etalons and could be significantly decreased by using a camera that minimizes diffraction artefacts. The sensor images displayed in (a-c) are highly saturated.

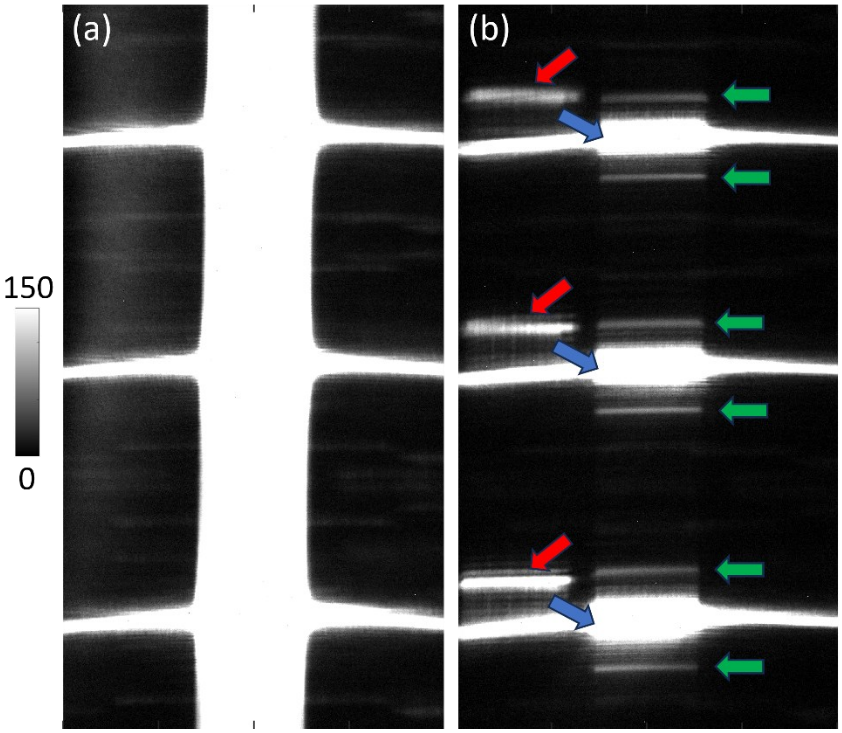

Fig. S5. Sensor images of the Brillouin signal from the milk dilution, without (a) vs. with (b) cascaded etalon. (a) and (b) are de-zoomed versions of the top and bottom panels of Fig. 5(h), respectively. In (b), the green arrows indicate the Brillouin signals, the blue arrows indicate the Rayleigh signal, and the red arrows indicate echoes of the main orders caused by back reflections from the cascaded etalon.

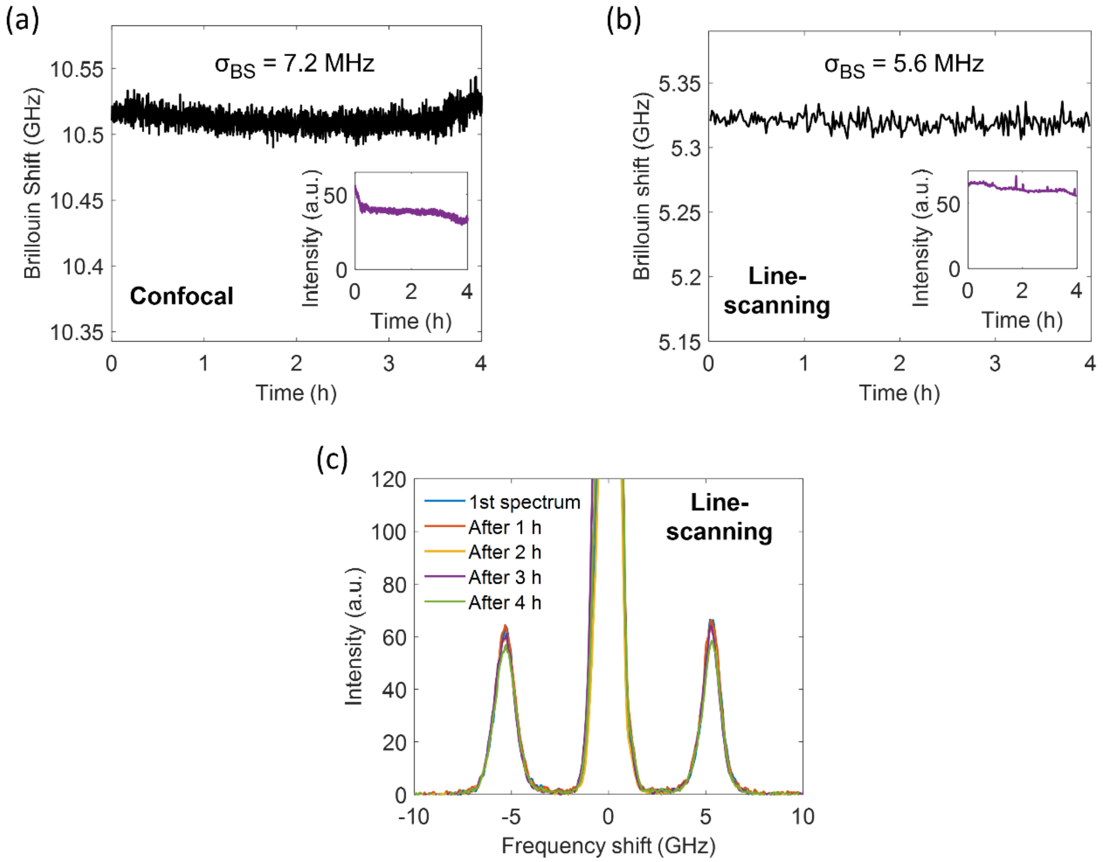

Fig. S6. Stability test. (a) Monitoring of the Brillouin shift of acrylic measured in the confocal setup, with the cascaded etalon included, over 4 hours. The standard deviation over the full period is 7.2 MHz. The inset shows the signal magnitude (averaged over the Stokes and anti-Stokes peaks) as a function of time. (b) Monitoring of the Brillouin shift of water (averaged over the central 25 μm of the line) measured in the line-scanning setup, with the cascaded etalon included, over 4 hours. The standard deviation over the full period is 5.6 MHz. The inset shows the signal magnitude (averaged over the Stokes and anti-Stokes peaks) as a function of time. (c) 5 spectra from the dataset analyzed in (b).
